# Dental Factors Associated With Oropharyngeal Dysphagia in Institutionalised Older Adults: A Systematic Review

**DOI:** 10.1111/ger.70034

**Published:** 2025-12-18

**Authors:** Raquel Soncini de Morais, Juliana Balbinot Hilgert, Fernando Neves Hugo, Rafaela Soares Rech

**Affiliations:** ^1^ Department of Preventive and Social Dentistry, Faculty of Dentistry Federal University of Rio Grande Do Sul Porto Alegre Brazil; ^2^ Department of Epidemiology and Health Promotion, College of Dentistry New York University New York New York USA; ^3^ Federal University of Health Sciences of Porto Alegre Porto Alegre Brazil

**Keywords:** aged, deglutition disorders, geriatric dentistry, oral health

## Abstract

**Background:**

Aging is associated with a decline in physiological functions. Dysphagia, a disorder among older adults, impairs feeding and is related to morphological and functional changes in the stomatognathic system, which worsen with age‐related physiological decline, particularly among institutionalised older adults.

**Objective:**

To systematically review the association between oral health conditions, dental status, xerostomia, and dysphagia among institutionalised older adults.

**Methods:**

This systematic review followed PRISMA guidelines and included a search of PubMed/MEDLINE and Embase for observational and randomised clinical trial studies published up to December 2024, with an update in January 2025.

**Results:**

From 1761 identified articles, 44 were reviewed, and 11 met the inclusion criteria. These studies employed subjective measures such as dysphagia questionnaires, the Eating Assessment Tool‐10 (EAT‐10), the Dysphagia Risk Evaluation Protocol (PARD), and interviews using the Minimum Data Set for Nursing Home Resident Assessment (MDS‐NH). Objective assessments included clinical evaluations, indirect and direct swallowing tests, and video analyses of feeding behaviour. Findings indicate that older individuals with fewer functional teeth, absent occlusal pairs, compromised natural teeth, gingivitis, or dental caries have a higher risk of dysphagia. In contrast, occlusal contact, natural teeth, or well‐fitted dentures are associated with reduced swallowing difficulties. Neither denture use alone nor oral hygiene showed a direct association with dysphagia. However, severe xerostomia and reduced salivary flow were associated with a higher risk of dysphagia.

**Conclusion:**

Dysphagia is associated with poor oral health and xerostomia in institutionalised older adults, though further research is needed to clarify these relationships.

## 1 Introduction

With the rapid growth of the older population and the preservation of natural teeth for longer periods, oral health problems among older adults living in long‐term care facilities are expected to increase significantly [1]. Systemic factors and limited access to dental care worsen this situation, contributing to malnutrition among frail older individuals. To address these challenges, long‐term care facilities should incorporate specific dental protocols into their health programs [2], establishing standardised parameters to assess the oral health of institutionalised older residents. Ideally, those assessments should be simple, objective, performed by a dentist, and consider aspects that affect the quality of life and general health, such as pain, inflammation, pathologies, and oral function [1]. However, several obstacles hinder effective oral health in long‐term care facilities, including resistance from residents, insufficient caregiver training, negative attitudes toward oral hygiene, and issues related to staffing, time constraints, and communication [3]. Additionally, admissions to long‐term care facilities are influenced by sociodemographic factors, health status, caregiver characteristics, and the use of medical and social services [4].

Oropharyngeal dysphagia is a common disorder that affects the oral preparatory, oral, and/or pharyngeal phases of swallowing, with diverse etiologies [5]. Although oropharyngeal dysphagia affects about 8% of the global population, equivalent to 590 million people, it remains underdiagnosed and undertreated in many healthcare centres. Oropharyngeal dysphagia is considered a geriatric syndrome due to its prevalence in older adults, multifactorial causes, association with comorbidities of poor prognosis, and the need for a multidimensional approach [6]. The mechanism underlying the initiation of swallowing during chewing solid foods is not yet fully understood, but it is known that oral factors, such as the lubrication of the food bolus, influence the duration of chewing and the onset of swallowing [7]. Oral health, in turn, is maintained through proper hygiene, but individuals with swallowing difficulties, especially older people with physical limitations, are at increased risk of developing oral problems, either due to difficulty chewing caused by tooth loss or the use of poorly fitted dentures [8].

A synthesis of available evidence regarding clinical and instrumental techniques for diagnosing dysphagia in older individuals, as well as the possible association with dental conditions in institutionalised older adults, is needed to clarify how these parameters are related. Therefore, this systematic review aims to assess whether oral health conditions are associated with oropharyngeal dysphagia in institutionalised older adults.

## 2 Methods

This systematic review was conducted in accordance with the Preferred Reporting Items for Systematic Reviews and Meta‐Analyses (PRISMA) guidelines and the protocol was registered in PROSPERO (CRD42022355765).

A comprehensive literature search was performed, including scientific articles and grey literature. Searches were carried out in MEDLINE (via PubMed) and EMBASE databases in February 2022, with the most recent update in January 2025. The search strategy was adapted for each database and is presented in Appendix S1. The search terms used individually or in combination included: ‘Older’, ‘Oral health’, and ‘Swallowing disorder.’ Articles published in any language were included in the study. We included all observational studies investigating the prevalence and risk factors of oropharyngeal dysphagia in institutionalised older individuals as well as randomised clinical trials from which relevant variables could be included. Due to the heterogeneity of swallowing assessment methods, we accepted clinical assessments, screening instruments, and instrumental evaluations.

For study selection, two reviewers (JBH, and RSM) independently screened titles and abstracts using the Rayyan platform. In cases of disagreement, a third researcher (RRS) acted as an arbitrator, participating in discussions until a consensus was reached regarding study inclusion or exclusion.

Data collection involved an initial exploratory reading of all selected material to confirm relevance, followed by a focused review of pertinent sections. Extracted data were recorded using a standardised research instrument. A standardised checklist, based on the eligibility criteria, was applied to each study identified in the initial search.

Analysis and interpretation of the results were carried out through an analytical reading to organise and synthesise data from the included sources. The categories that emerged were analysed and discussed based on the relevant theoretical framework. The risk of bias for each included study was independently assessed by one reviewer (RSM) using the Joanna Briggs Institute Critical Appraisal Checklist, with specific criteria applied for observational (cross‐sectional) and interventional studies (randomised clinical trials). Each study was categorised according to the percentage of positive responses: high risk of bias (≤ 49% ‘yes’ responses), moderate risk (50%–69%), and low risk (≥ 70%).

## 3 Results

**FIGURE 1:**
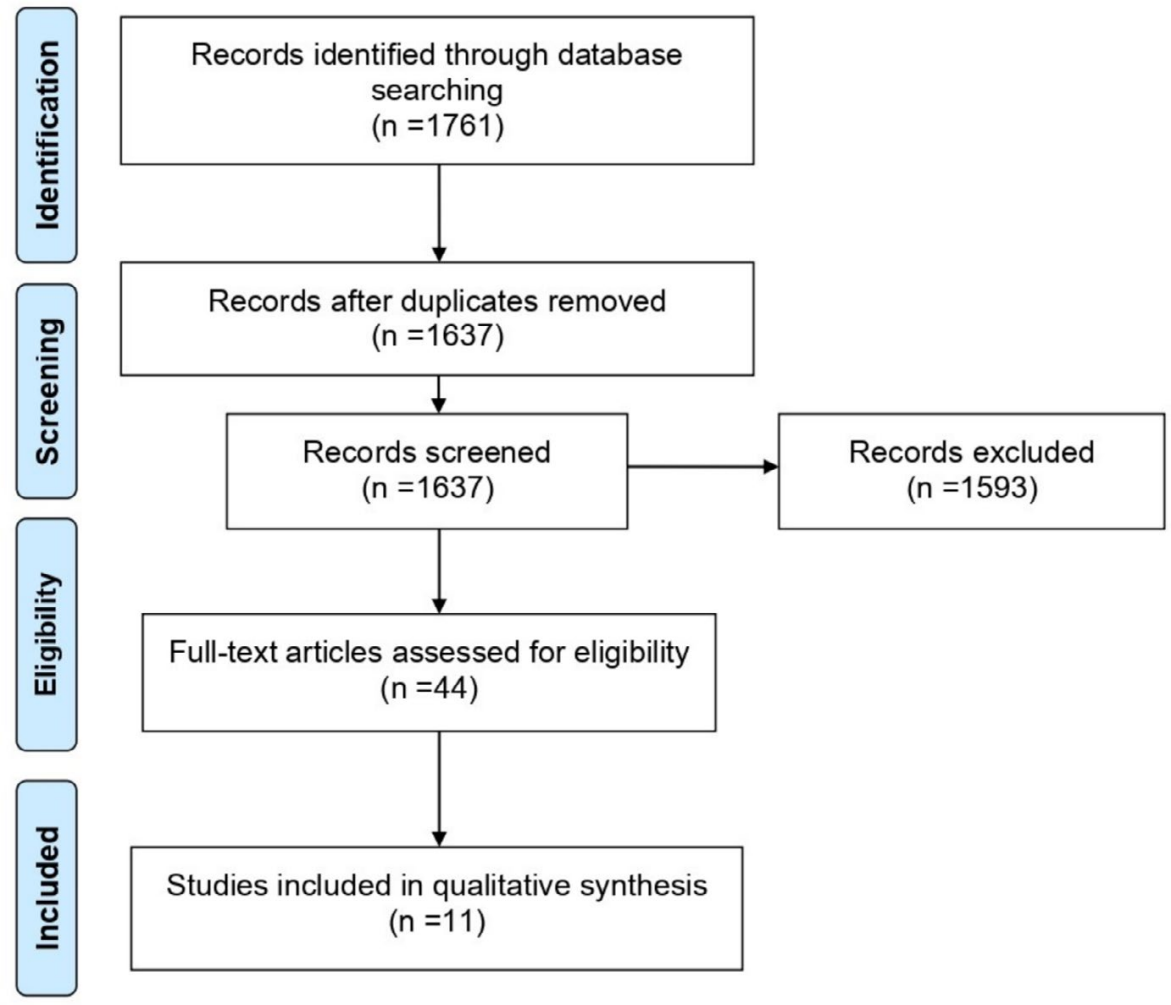
Flow diagram of study selection. [Colour figure can be viewed at wileyonlinelibrary.com]

The search strategy identified 1761 studies. After screening titles and abstracts, 1593 publications that did not meet the eligibility criteria were excluded, leaving 44 articles for full‐text reading. Of these, 11 studies (ten cross‐sectional studies [9, 10, 11, 12, 13, 14, 15, 16, 17, 18] and one randomised clinical trial [19]) were included in the analysis. The reasons for exclusion of the remaining full‐text articles are detailed in Appendix S2. The PRISMA flowchart (Figure 1) illustrates the selection process and the reasons for exclusion at each stage.

The following information was extracted and synthesised: study identification (author, year, journal, country), study design, follow‐up period, participant details (number of participants, age; Table 1), comparison/intervention groups (oral health assessment; Table 2), outcome analysis (subjective and objective measures for oropharyngeal dysphagia evaluation; Table 3), and results (Table 4).

**TABLE 1:** Characteristics of the included studies.

Study	Journal	Country	Study design	Follow up/Collection period	Sample size: *n* (F/M)	Age mean ±SD
						Age group: *n* (%)
Izumi et al. (2021) [19]	Journal of Oral Rehabilitation	Japan	Randomised controlled trial	October 2017 to May 2019	24 (21/3)	65 years or older
Miura et al. (2004) [9]	Nippon Ronen Igakkai Zasshi Japanese Journal of Geriatrics	Japan	Cross‐sectional	December 2002 to March 2003	92 (62/30)	80.6 ± 8.6
Bomfim et al. (2013) [10]	CoDAS (Communication Disorders, Audiology and Swallowing)	Brazil	Cross‐sectional	August to December 2007	30 elder women	83.7 ± 10.6
Saintrain and Gonçalves (2013) [11]	Gerodontology	Brazil	Cross‐sectional	September to October 2007	68 (33/35)	70.4 ± 7.3
Okabe et al. (2017) [12]	Journal of Oral Rehabilitation	Japan	Cross‐sectional	February to June 2014	238 (186/52)	86.6 ± 6.5
Brochier et al. (2018) [13]	Gerodontology	Brazil	Cross‐sectional	April to October 2016	115 (77/38)	60–70: 22 (19.1) 71–80: 42 (36.6) 81 >: 51 (44.3)
Rech et al. (2018) [14]	Oral Diseases	Brazil	Cross‐sectional	2016	123 (81/42)	73.5 ± 8.9
Zorawna et al. (2023) [15]	Journal of Oral Rehabilitation	Finland	Cross‐sectional	September 2017 to February 2019	338 (244/94)	Group 1: 80 ± 8; Group 2:81 ± 8; Group 3: 82 ± 8; Group 4:83 ± 9
Yatabe et al. (2018) [16]	Gerodontology	Japan	Cross‐sectional	February to June 2014	236 (184/52)	87.7 ± 6.4 (dentate); 89.0 ± 7.1 (edentulous)
Wang et al. (2012) [17]	Geriatric Nursing	China	Cross‐sectional	x	781 (308/473)	79.4 ± 10.3
Chen et al. (2020) [18]	BMC Geriatrics	China	Cross‐sectional	May to July 2019	775 (470/305)	81.3 ± 9.3

**TABLE 2:** Characteristics of the included studies (comparison/intervention groups).

Study	Subjective	Objective
Izumi et al. (2021) [19]		A dental hygienist performed oral examinations, assessing posterior occlusal support by counting functional tooth units, with a maximum score of 12
Miura et al. (2004) [9]	The frequency of dental and oral cleaning was assessed using the number of times per day	Oral cleanliness was assessed by counting anaerobic bacteria and streptococci in dental plaque from the upper molars, with results expressed as a common logarithm
Bomfim et al. (2013) [10]	The items related to eating dynamics were recorded during or near mealtimes by trained students, following a previously described protocol and criteria	
Saintrain and Gonçalves (2013) [11]		Salivary pH and flow were measured before and after stimulation by chewing, using indicator paper and saliva collection for comparison
Okabe et al. (2017) [12]		A dentist assessed the number of teeth and posterior occlusion of the participants, classifying them by the number of natural teeth. Occlusion was measured in functional tooth units (FTUs) to predict the risk of dysphagia
Brochier et al. (2018) [13]	Xerostomia was assessed using an 11‐item inventory that measures the frequency of dry mouth symptoms, with scores grouped into quartiles for analysis	A dental examination assessed teeth, prostheses, and occlusal pairs, checking the type, retention, stability, risk of injury, and aesthetics of the prostheses, which were classified according to their fit. Oral health was evaluated by a dentist according to World Health Organization (WHO) criteria
Rech et al. (2018) [14]		A dentist assessed oral functionality according to World Health Organization (WHO) criteria, classifying it as functional, partially functional, or non‐functional, based on tooth presence and prosthesis fit
Zorawna et al. (2023) [15]		The oral examination assessed the number of teeth, the use and condition of dentures, and occlusal support through contact units, considering only active denture users
Yatabe et al. (2018) [16]		A dentist assessed the number of teeth and posterior occlusal support, classifying participants as dentate (≥ 1 tooth) or edentulous based on the presence or absence of tooth contact
Wang et al. (2012) [17]	Residents' needs were assessed using the Chinese version of the Minimum Data Set for Resident Assessment and Care Screening in Nursing Homes (MDS‐NH), gathering personal, institutional, functional, and health data	
Chen et al. (2020) [18]	A self‐designed questionnaire collected demographic data, health status, clinical history, diet, medication, and dental condition of the participants	

**TABLE 3:** Characteristics of the included studies (subjective and objective measures for the evaluation of oropharyngeal dysphagia).

Study	Subjective	Objective
Izumi et al. (2021) [19]		Swallowing function was assessed using the Modified Water Swallowing Test (MWST)
Miura et al. (2004) [9]	Items to assess eating/swallowing disorders were developed by medical institutions, based on previous questionnaires	
Bomfim et al. (2013) [10]	Data were collected from medical records regarding identification, length of institutionalisation, diagnoses, medications, cognitive status, and independence Swallowing assessment, performed by a speech‐language pathologist before the filming of meals, recorded signs of oropharyngeal dysphagia according to the Pathology Risk Assessment Protocol for Dysphagia (PARD)	Data were collected through medical record analysis and by filming each older woman's lunch, which was carried out by trained students. The analysis of behavioural aspects and feeding dependence was performed by a specialist speech‐language pathologist
Saintrain and Gonçalves (2013) [11]	A closed‐ended questionnaire was administered to the older adults to collect demographic data, diseases, medication use, and oral discomfort	
Okabe et al. (2017) [12]		A dental hygienist assessed swallowing using a Modified Water Swallowing Test (MWST), repeated twice if the score was ≥ 4; a score ≤ 3 indicated a risk of dysphagia
Brochier et al. (2018) [13]		The clinical swallowing assessment had two phases: indirect and direct tests with foods of different consistencies, observing signs of dysphagia. Any abnormality indicated dysphagia
Rech et al. (2018) [14]		The swallowing assessment included both indirect and direct tests with three food consistencies, analysing various clinical signs and using cervical auscultation. Dysphagia was diagnosed if any abnormality was detected
Zorawna et al. (2023) [15]	In the FINORAL study, demographic data, diagnoses, and medications were collected from medical records, and nurses used a standardised questionnaire to record swallowing difficulties	
Yatabe et al. (2018) [16]		A dentist used the Modified Water Swallowing Test (MWST) for dysphagia screening. The test involved swallowing 3 mL of cold water and scoring the swallowing. Scores of 4 or 5 were repeated twice, with the lowest score used. A score of ≤ 3 indicated a risk of dysphagia
Wang et al. (2012) [17]	The participants were evaluated using the Chinese version of the Minimum Data Set for Resident Assessment and Care Screening in Nursing Homes (MDS‐NH) and quality of life tools	
Chen et al. (2020) [18]	The Eating Assessment Tool‐10 (EAT‐10) scale is a quick 10‐question questionnaire to identify the risk of dysphagia, with a score of ≥ 3 indicating risk	

**TABLE 4:** Characteristics of the included studies (results).

Study	Results
Izumi et al. (2021) [19]	The swallowing function, as assessed by the Modified Water Swallowing Test (MWST), did not significantly change between baseline and the end of the follow‐up period in either group. All participants could continue to ingest food during the follow‐up period
Miura et al. (2004) [9]	The presence or absence of risk factors for eating/swallowing disorders was not significant for the number of mouths cleanings, the total number of anaerobic bacteria and the total number of streptococci in dental plaque. No significant association was observed
Bomfim et al. (2013) [10]	In the group of older women with signs suggestive of dysphagia, there was a higher use of medication, a lower occurrence of depression, a greater number of teeth, and more alterations in eating dynamics
Saintrain and Gonçalves (2013) [11]	There was statistical significance with respect to salivary flow without stimulation and dry mouth (*p* = 0.018) and difficulty in swallowing (*p* = 0.046)
Okabe et al. (2017) [12]	The subjects with dysphagia risk had a significantly lower number of total functional tooth units (FTUs) than those without dysphagia risk
Brochier et al. (2018) [13]	Older adults with no occlusal pairs (PR = 1.52 (95% CI = 1.02–2.40)) had higher prevalence of oropharyngeal dysphagia compared to those with 8 to 14 mixed pairs. No significant association was found with 1 to 7 compared to those with 8 to 14 mixed pairs. Additionally, higher xerostomia scores were positively associated with a greater prevalence of oropharyngeal dysphagia (PR = 2.86 [95% CI = 1.58–5.16]) for 30 to 50 score and (PR = 3.01 [95% CI = 1.67–5.42]) for 20 to 29 score
Rech et al. (2018) [14]	Individuals who presented a non‐functional oral status (PR = 1.61; 95% CI 1.02–2.54) presented a higher frequency of dysphagia
Zorawna et al. (2023) [15]	The study revealed that removable denture users experienced swallowing difficulties less frequently than those without complete upper dentures
Yatabe et al. (2018) [16]	The proportions of participants at risk for dysphagia were 18.9% in the dentulous group and 15.2% in the edentulous group, respectively. Additionally, 43.2% of participants in the dentulous group and 40.8% in the edentulous group had oral intake with restrictions
Wang et al. (2012) [17]	Significantly fewer residents with problems with chewing and swallowing (PCS) still had some or all their natural teeth compared with those without problems with chewing and swallowing (PCS) (*p* < 0.001). They also had more broken, loose, or decayed teeth (*p* = 0.043) and more inflamed gums (*p* < 0.001). Significantly more residents with problems with chewing and swallowing (PCS) suffered from oral pain than did those without problems with chewing and swallowing (PCS) (*p* = 0.033)
Chen et al. (2020) [18]	In the univariate analysis, having natural or artificial teeth was associated with a lower prevalence of dysphagia, whereas the presence of dental caries was more frequent among individuals with dysphagia. This association, however, was not maintained after adjustment

The included studies were conducted in Brazil, Japan, China, and Finland and were published between 2004 and 2023, all carried out in Long‐Term Care Institutions for Older Adults [9, 10, 11, 12, 13, 14, 15, 16, 17, 18, 19]. In total, 2820 institutionalised older individuals participated in the 11 selected studies, with an average age of 87 years. The number of participants per study ranged from 24 [19] to 781 [17].

**FIGURE 2:**
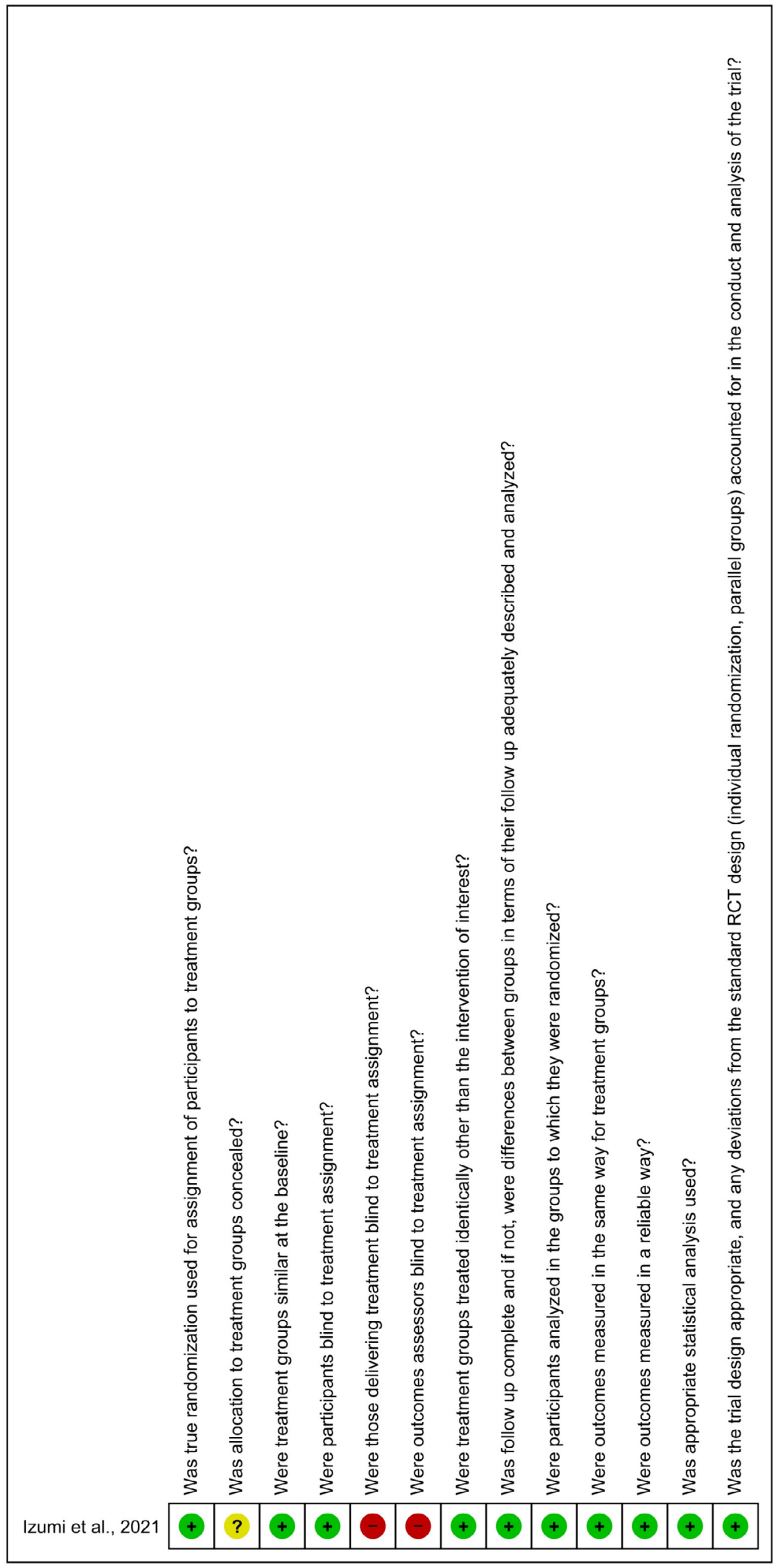
Results of the risk of bias assessment of the included studies (randomised clinical trial). [Colour figure can be viewed at wileyonlinelibrary.com]

**FIGURE 3:**
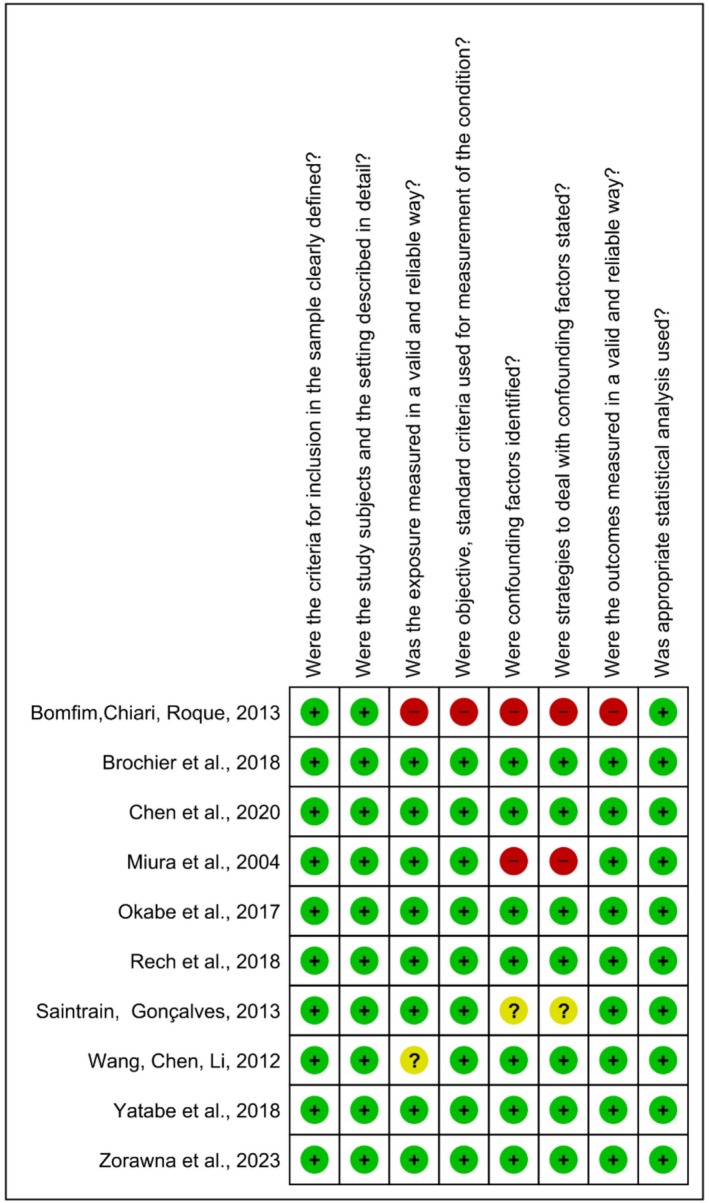
Results of the risk of bias assessment of the included studies (cross‐sectional). [Colour figure can be viewed at wileyonlinelibrary.com]

The risk of bias assessments (Figures 2 and 3) were performed using Review Manager Software 5.3, generated by Review Manager (v. 5.4.1). Of the 11 included studies, most were classified as having a low risk of bias, comprising 9 cross‐sectional studies [9, 11, 12, 13, 14, 15, 16, 17, 18] and 1 randomised clinical trial [19]. One cross‐sectional study was rated as having a high risk of bias [10]. In the randomised clinical trial, the item most frequently marked as ‘no’ was related to the blinding of the treatment administrator and outcome assessor. Among the cross‐sectional studies, the most common sources of high risk bias were the lack of identification of confounding factors and the absence of strategies to manage them.

### 3.1 Outcome Measures

Outcome measures varied among the studies and included both subjective and objective methods. Subjective assessments comprised questionnaires developed by health institutions to evaluate symptoms of eating and swallowing disorders [9], questionnaires with closed‐ended questions about swallowing difficulties [11], and the FINORAL study questionnaire that included yes/no questions about swallowing problems [15]. The Eating Assessment Tool‐10 (EAT‐10), a dysphagia screening tool, 10 items scored from 0 (no problem) to 4 (severe problem), was used to classify participants with a total score ≥ 3 as having dysphagia [18]. Signs suggestive of oropharyngeal dysphagia were also recorded from medical records using the Speech‐Language Pathology Risk Assessment Protocol for Dysphagia (PARD), previously conducted by a speech‐language pathologist [10]. Furthermore, interviews were conducted using the Chinese version of the Minimum Data Set for Resident Assessment and Care Screening in Nursing Homes (MDS‐NH) to evaluate chewing or swallowing problems [17].

Objective measures of oropharyngeal dysphagia were obtained through clinical assessments. Swallowing function was evaluated in some studies using the modified water swallowing test (MWST), in which three millilitres of cold water are injected onto the floor of the participant's mouth using a 5 mL syringe, and the swallowing process is then assessed [12, 16, 19]. In two studies, the swallowing evaluation was divided into indirect tests (e.g., saliva swallowing, forced coughing, anatomical examination) and direct tests (e.g., swallowing of liquid, semi‐solid, and solid foods [13] as well as foods with pasty, liquid, and solid consistencies) [14]. During the direct tests, signs of laryngotracheal penetration or aspiration were observed, such as masticatory efficiency, bolus formation time, coughing, choking, food retention in the throat, voice changes, and feeding discomfort. The absence of dysphagia required normality in all evaluated items [13, 14]. One study assessed signs suggestive of dysphagia through a video recording of each participant, documenting ‘attitudinal and behavioral aspects related to feeding’ and ‘feeding dependence and required assistance,’ with analysis conducted by a speech‐language pathologist [10].

To describe the relationship between oropharyngeal dysphagia and oral health, the results were organised according to oral health measures and potential associations with dysphagia were discussed.

#### 3.1.1 Functional Dental Units

Three studies investigated occlusal pairs as predictors of dysphagia. Participants at risk of dysphagia had significantly fewer functional dental units than those without dysphagia risk [12]. Older adults without occlusal pairs also showed a higher prevalence of oropharyngeal dysphagia than those with 8 to 14 mixed pairs (natural and artificial) [13]. Additionally, individuals with mixed dentition (natural teeth, with or without removable prostheses) and occlusal contact units showed a lower frequency of swallowing difficulties than those with teeth without occlusal contact, edentulous individuals, or users of a complete denture in only one jaw [15].

#### 3.1.2 Oral Care

A randomised clinical trial evaluating the effectiveness of tongue cleaning on swallowing function found no significant changes between baseline and the end of the follow‐up period in either the intervention or control groups [19]. No relationship was found between the presence or absence of possible determinants of eating/swallowing disorders and the number of daily oral cleanings, nor with the total number of anaerobic bacteria or streptococci in dental plaque [9]. Similarly, another cross‐sectional study found no association between swallowing problems and the presence of food residues in the mouth before bedtime, the frequency of daily cleaning of teeth/dentures, or daily oral care [17]. No significant differences in oral hygiene were observed between older women with and without signs suggestive of oropharyngeal dysphagia [10].

#### 3.1.3 Oral Condition

Individuals with swallowing problems more frequently had compromised natural teeth (broken, loose, or decayed), gingivitis, and oral pain than those without these issues [17]. A higher prevalence of dysphagia was also associated with a greater number of dental caries [18]. On the other hand, another study found no significant differences in dental conservation status (adequate or inadequate) between older women with and without signs suggestive of oropharyngeal dysphagia [10].

#### 3.1.4 Number of Teeth

Individuals with more natural teeth had a lower likelihood of experiencing swallowing problems [17]. The probability of dysphagia was 18.9% in the dentate group (≥ 1 tooth) and 15.2% in the edentulous group [16]. However, another study indicated that the number of teeth was higher in women with signs suggestive of oropharyngeal dysphagia [10].

Oral functionality, assessed based on the presence of teeth and the use and fit of dental prostheses (retention, stability, and oral tissue injury), was classified as functional, partially functional, or non‐functional. No higher prevalence of dysphagia was observed in individuals with non‐functional oral health status [14].

#### 3.1.5 Dental Prostheses

No significant differences were found between the use of dental prostheses and signs of oropharyngeal dysphagia [10], swallowing problems [17], or the prevalence of dysphagia considering prosthesis fit [13].

#### 3.1.6 Xerostomia and Salivary Problems

High scores on the Xerostomia Inventory (XI), indicative of more severe chronic xerostomia symptoms, were associated with a higher prevalence of oropharyngeal dysphagia [13]. In pH and salivary flow rate tests, both with and without stimulation, a significant association was observed between unstimulated salivary flow and the frequency distribution of unstimulated salivary pH to swallowing difficulty [11].

## 4 Discussion

This systematic review demonstrates an association between the number of occlusal units, the presence of functional teeth, and swallowing ability in institutionalised older adults. Deterioration in oral health, particularly tooth loss, and changes in occlusal units are associated with a higher frequency of symptoms of oropharyngeal dysphagia, underscoring the importance of oral health as a significant factor in managing swallowing disorders in this population. The comparison with the institutionalised older population, an underexplored sample, reveals new perspectives for the literature, highlighting the need for integrated strategies that consider oral health as a relevant factor in the management of oropharyngeal dysphagia in this specific population.

The focus on institutionalised older adults, an often‐underrepresented group in literature, provides new perspectives and highlights the need for integrated strategies that address oral health as a central factor in the prevention and management of oropharyngeal dysphagia in long‐term care settings. Oropharyngeal dysphagia is highly prevalent among older adults, affecting approximately 30% of community‐dwelling individuals, nearly 50% of geriatric patients, and more than 50% of nursing home residents [20]. However, prevalence estimates in institutionalised older persons vary widely (5.4% to 83.7%), mainly due to methodological heterogeneity across studies [21]. This variability emphasises the importance of ongoing investigation and standardised monitoring of oropharyngeal dysphagia in this population [22].

While previous reviews have examined the relationship between oral health and dysphagia, they present limitations. One review identified a high prevalence of dysphagia among institutionalised older adults but focused exclusively on dentition‐related assessments [23]. Another review suggested a possible association between oral health and dysphagia; still, it did not include institutionalised populations and emphasised the absence of standardised indicators to assess the broader impact on health [24]. This gap reinforces the importance of targeting this vulnerable population, which is particularly susceptible to rapid declines in oral health due to comorbidities, polypharmacy, and decreased self‐care capacity.

The trend toward increased longevity and retention of natural teeth means that oral health problems among institutionalised older people are also expected to grow quickly [1], being directly related to general health and quality of life. Common risk factors, such as chronic diseases and reduced self‐care ability, further exacerbate oral conditions [25]. Tooth loss or absence of functional teeth impairs chewing efficiency, making food preparation and swallowing more difficult [26].

Consistent with the findings of this review, other studies have shown that poor oral health, including tooth loss, contributes to swallowing problems. A retrospective cohort study showed that in older adults with dysphagia, poor oral health, including the loss of natural teeth, was identified as a factor that hinders the improvement of swallowing function [27]. Moreover, the prevalence of self‐reported dysphagia is higher among older adults with a lower number of permanent teeth. These findings highlight the need for further research on how the number, location, and function of teeth influence the occurrence of dysphagia in this population [26].

Xerostomia also emerged as a significant factor associated with dysphagia. Notably, dry mouth is a frequent complaint among individuals with swallowing disorders, and its prevalence tends to be higher with advancing age and among institutionalised individuals. Other studies also highlight that oral dryness is more related to dysphagia in adults over 50 years old [28]. That reduced salivary flow hinders the improvement of swallowing in older individuals with oropharyngeal dysphagia [27, 29]. Saliva plays a crucial role in the initial phase of swallowing by facilitating mastication, enzymatic digestion, and the transport of the food bolus through the oropharynx, as well as protecting the mucosa and aiding in taste perception [30]. Xerostomia may exacerbate swallowing difficulties by impairing bolus formation and lubrication and reducing the ability to moisten and bind food particles, which are essential for forming a cohesive and safe‐to‐swallow bolus [31].

Interestingly, our review found no direct association between oral care practices and dysphagia, which aligns with previous systematic reviews reporting limited evidence regarding the effectiveness of regular oral care or oral disinfection in reducing aspiration pneumonia among individuals with swallowing disorders [27]. Nonetheless, twice‐daily oral hygiene using antibacterial toothpaste is the most promising intervention for improving oral health [32]. Similarly, no association was identified between the use of dental prostheses and dysphagia. However, a retrospective cohort study suggests that ill‐fitting prostheses may hinder the improvement of swallowing function in older individuals with dysphagia, indicating that compromised oral conditions can negatively impact swallowing by limiting the variety and safety of food intake [27]. Regarding dysphagia assessment, it is essential to adopt systematic approaches to identify individuals at risk. Due to their practicality, objective assessment methods, such as the Modified Water Swallowing Test (MWST), are commonly employed in long‐term care institutions. However, they present limitations when compared to gold‐standard instrumental assessments like videofluoroscopy or fiberoptic endoscopic evaluation of swallowing (FEES). Given that many long‐term care institution residents are unable to reliably self‐report swallowing difficulties, our review identified that validated screening tools such as the Gugging Swallowing Screen (GUSS) and the Standardised Swallowing Assessment (SSA) are recommended for use by nursing staff to enable early detection and prevent complications [33, 34].

Our findings reinforce that early diagnosis and appropriate management of dysphagia should be a priority in long‐term care institutions [35]. Clinical swallowing assessment encompasses a set of procedures that can be adapted to different populations. It aims to identify potential causes of dysphagia, assess swallowing safety and aspiration risk, determine whether oral feeding is appropriate or alternative methods are needed, guide the need for further instrumental assessments, and establish baseline data for follow‐up [36, 37].

Despite the multidimensional nature of oropharyngeal dysphagia, our review found that most non‐instrumental clinical assessments focus on isolated subdomains, and there is currently no international consensus on a gold‐standard clinical tool for non‐instrumental dysphagia assessment [38]. Additionally, while cervical auscultation is sometimes used as an adjunct to clinical evaluation by capturing acoustic signals related to swallowing [36, 39], the evidence indicates that its reliability is insufficient when used as a standalone diagnostic method [40].

The systematic review presents some limitations. The scarcity of studies in long‐term care facilities weakens the evidence's applicability. The lack of standardisation in outcome assessment limits comparisons and may result in over‐ or underestimation of the reported measures of association, restricting the possibility of conducting a meta‐analysis. Future research should prioritise standardising methodologies and careful planning.

## 5 Conclusion

Aging leads to changes in the stomatognathic system that impact swallowing, especially among institutionalised older adults, who are generally more vulnerable. The frequency of dysphagia in this group is high, though it varies widely due to differences in diagnostic methods.

Factors such as a reduced number of functional teeth, absence of occlusal contact, presence of oral diseases, and xerostomia have been associated with a higher frequency of dysphagia symptoms, whereas characteristics such as functional adaptation and the use of well‐fitted dentures have been related to better swallowing performance. However, inconsistencies in the literature regarding the relationship between dental status and dysphagia highlight the need for further research to clarify these aspects.

## Author Contributions

R.S.R., J.B.H., and F.N.H. conceived and designed the protocol for the systematic review. R.S.M., J.B.H., and R.S.R. independently participated in the study selection process, applying predefined criteria. Data extraction and interpretation of the results were carried out by R.S.M. Data analysis and preparation of the initial manuscript draft were performed by R.S.M., R.S.R., and J.B.H. F.N.H. provided strategic guidance throughout the process, especially regarding the methodological design of the review. All authors contributed and approved the final version of the manuscript, ensuring shared responsibility for the content presented.

## Funding

This study was financed in part by the Coordenação de Aperfeiçoamento de Pessoal de Nível Superior—Brasil (CAPES).

## Ethics Statement

The authors have nothing to report.

## Consent

All authors have read and approved the final version of the manuscript and consent to its publication.

## Conflicts of Interest

The authors declare no conflicts of interest.

## Supporting information


**Appendix S1:** Search strategy used in PUBMED/MEDLINE.

**Appendix S2:** Reasons for exclusion of full‐text articles.

## Data Availability

The data and materials supporting this manuscript are included in the article and Supporting Information files.
