# Supplementary material for: Dental Factors Associated With Oropharyngeal Dysphagia in Institutionalised Older Adults: A Systematic Review
Source: Gerodontology. 2025 Dec 18;43(2):159–71. doi: 10.1111/ger.70034 (PMC13140015; doi:10.1111/ger.70034)
Supplement: Supplementary file 1 — Appendix S1: Search strategy used in PUBMED/MEDLINE. [file GER-43-159-s001.docx]

APPENDIX 1

Search strategy used in PUBMED/MEDLINE

| **Index** | **Strategy** | **Results** |
| --- | --- | --- |
| #1 **Older** | "Aged"[Mesh] OR Aged OR ((older* OR old* OR aged) AND (person* OR adult* OR people OR patient*)) OR "Aging"[Mesh] OR aging OR ageing OR senescence OR senescent | 6,686,666 |
| #2 **Restrictor** 1 | "Frail Elderly"[Mesh] OR "Frail Elderly" OR ((Frail OR “Functionally-Impaired” OR “Functionally Impaired”) AND (Aged OR ((older* OR old* OR aged) AND (person* OR adult* OR people OR patient*)))) | 28,939 |
| #3 **Restrictor** 2 | "Nursing Homes"[Mesh] OR ((Nursing OR “extended care” OR convalescence) AND (Home* OR facility)) | 127,341 |
| #4 **Restrictor** 3 | "long-term care"[mesh] OR “long-term care” OR “long term care” | 43,598 |
| #5 **Restrictor** 4 | "geriatric nursing"[mesh] OR (geriatric AND nursing*) | 33,723 |
| #6 **Restrictor** 5 | "Institutionalization"[Mesh] OR "Institutionalization" OR (Institutionaliz* AND Person*) OR (independent elders) | 16,808 |
| #7 **Patients** | #1 AND (#2 OR #3 OR #4 OR #5 OR #6) | 125,106 |
| #8 **Oral condition** | ((((oral health) OR (poor oral health)) OR (oral health[MeSH Terms])) OR (dental health)) OR (dental care)) OR (oral hygiene[MeSH Terms])) OR (oral factors)) | 519,326 |
| #9 **Oral condition** | ((((((((((prosthodontic[MeSH Terms]) OR (dentures[MeSH Terms])) OR (denture)) OR (complete denture[MeSH Terms])) OR (dental prostheses[MeSH Terms])) OR (mandibular prosthesis[MeSH Terms])) OR (maxillary prosthesis[MeSH Terms])) OR (mouth rehabilitation[MeSH Terms])) OR (partial denture, removable[MeSH Terms])) OR (((((((((((((dentures) OR (oral rehabilitation)) OR (conventional denture)) OR (dental flipper)) OR (tooth prosthesis)) OR (swing-lock)) OR (rpd (denture))) OR (removable partial dentures)) OR (conventional complete dentures)) OR (full mouth rehabilitation)) OR (removable denture)) OR (dental prostheses)) OR (dental prosthesis)) OR (prosthetic rehabilitation)) OR (dentures) | 168,071 |
| #10 **Oral condition** | (((((((((((((((((((((((((edentulous mandible) OR (edentulous patient rehabilitated)) OR (totally edentulous patients)) OR (totally edentulous)) OR (edentulous treatment)) OR (edentulousness)) OR (edentulous state)) OR (edentulous patient)) OR (edentulous jaw)) OR (maxillary edentulous)) OR (toothlessness)) OR (edentate)) OR (edentulism)) OR (tooth loss)) OR (toothless)) OR (edentulous)) OR (edentulous jaw[MeSH Terms])) OR (edentulous mouth[MeSH Terms])) OR (tooth loss[MeSH Terms])) OR (toothless mouth[MeSH Terms])) OR (completely edentulous)) ) OR (fully edentulous)) OR (total toothlessness)) OR (toothless patient)) OR (dental loss) | 57,743 |
| #11 **Oral condition** | ((((((((((((((malocclusion[MeSH Terms]) OR (bite, open[MeSH Terms])) OR (dental malocclusion)) OR (jaw malocclusion)) OR (jaw occlusion disorder)) OR (malocclusion)) OR (occlusion disorder, jaw)) OR (occlusion, mal)) OR (open bite)) OR (overbite)) OR (tooth malocclusion)) OR (maxillary jaw relationship)) OR (occlusal contacts)) OR (occlusal dental)) OR (occlusal status) | 58,431 |
| #12 **Oral condition** | ((((((((((((((xerostomia) OR (xerostomia treatment)) OR (dry mouth)) OR (mouth dryness)) OR (oral dryness)) OR (saliva flow)) OR (saliva release)) OR (saliva secretion)) OR (salivary flow)) OR (salivary secretion)) OR (salivary gland secretion)) OR (xerostomia[MeSH Terms])) OR (salivation[MeSH Terms])) OR (hyposalivation[MeSH Terms])) OR (hyposalivation) | 98,882 |
| #13 **Oral condition** | #8 OR #9 OR #10 OR #11 OR #12 | 773,834 |
| #14 **Institutionalized older** + **Oral condition** | #7 AND #13 | 4,627 |
| #15 **Outcome** | "Deglutition Disorders"[Mesh] OR (swallow* OR deglutition OR Oropharyngeal AND (problem* OR disorder* OR impairment* OR difficult* OR function OR dysphagia) OR dysphagia)) | 108,657 |
| #16 **Search** | #15 AND #16 | 495 |

Search strategy used in EMBASE

| **Index** | **Strategy** | **Results** |
| --- | --- | --- |
| #1 **Older** | 'aged'/exp OR aged OR 'aging'/exp OR aging OR ageing OR 'senescence'/exp OR 'senescence' OR senesense | 6,743,492 |
| #2 **Older** | (elder* OR old* OR aged) AND (person* OR adult* OR people OR patient*) | 6,573,622 |
| **#3 Older** | #1 OR #2 | 7,940,079 |
| #4 **Restrictor** 1 | 'frail elderly'/exp OR 'frail elderly' | 15,556 |
| #5 **Restrictor** 2 | (frail OR 'functionally-impaired' OR 'functionally impaired') AND (aged OR ((older* OR old* OR aged) AND (person* OR adult* OR people OR patient*))) | 33,690 |
| #6 **Restrictor** 3 | 'nursing home'/exp | 66,487 |
| #7 **Restrictor** 4 | (nursing OR 'extended care' OR convalescence) AND (home* OR facility) | 396,852 |
| #8 **Restrictor** 5 | 'long term care'/exp | 2,465,651 |
| #9 **Restrictor** 6 | ('long-term' OR 'long term') AND care | 397,761 |
| #10 **Restrictor** 7 | 'geriatric nursing'/exp | 13,217 |
| #11 **Restrictor** 8 | geriatric AND nursing* | 82,611 |
| #12 **Restrictor** 9 | 'institutionalization'/exp | 9,613 |
| #13 **Restrictor** 10 | 'institutionalization' OR (institutionaliz* AND person*) | 16,554 |
| #14 **Restrictors** | #4 OR #5 OR #6 OR #7 OR #8 OR #9 OR #10 OR #11 OR #12 OR #13 | 3,061,741 |
| #15 **Institutionalized older** | #3 AND #14 | 1,334,408 |
| #16 **Oral condition** | 'oral health' | 66,597 |
| #17 **Oral condition** | 'dental loss' OR 'tooth loss' | 6,935 |
| #18 **Oral condition** | 'oral rehabilitation'/exp OR 'denture'/exp OR 'conventional denture' OR 'dental flipper' OR 'dental flippers' OR 'denture' OR 'dentures' OR 'tooth prosthesis'/exp OR 'dental prostheses' OR 'dental prosthesis' OR 'denture prosthesis' OR 'prostheses, dental' OR 'prosthesis, dental' OR 'prosthesis, tooth' OR 'tooth prosthesis' OR 'removable partial denture'/exp OR 'rpd (denture)' OR 'swing-lock' OR 'denture, partial, removable' OR 'partial denture, removable' OR 'partial dentures, removable' OR 'removable partial denture' OR 'removable partial dentures' | 116,449 |
| #19 **Oral condition** | 'edentulism'/exp OR 'completely edentulous' OR 'edentulism' OR 'edentulous mouth' OR 'edentulous patient' OR 'edentulous state' OR 'edentulousness' OR 'fully edentulous' OR 'mouth, edentulous' OR 'toothless mouth' OR 'toothless patient' OR 'total toothlessness' | 13,382 |
| #20 **Oral condition** | 'malocclusion'/exp OR 'dental malocclusion' OR 'jaw malocclusion' OR 'jaw occlusion disorder' OR 'malocclusion' OR 'occlusion disorder, jaw' OR 'occlusion, mal' OR 'open bite' OR 'overbite' OR 'tooth malocclusion' | 41,500 |
| #21 **Oral condition** | 'xerostomia'/exp OR 'dry mouth' OR 'mouth dryness' OR 'oral dryness' OR 'xerostomia' OR 'xerostomy' OR 'zerostomiasis' OR 'salivation'/exp OR 'saliva flow' OR 'saliva release' OR 'saliva secretion' OR 'salivary flow' OR 'salivary secretion' OR 'salivation' OR 'salivary gland secretion' | 65,588 |
| #22 **Oral condition** | #16 OR #17 OR #18 OR #19 OR #20 OR #21 | 283,087 |
| #23 **Institutionalized older** + **Oral condition** | #3 AND #14 AND #22 | 15,493 |
| #24 **Dysphagia** 1 | ‘dysphagia'/exp OR 'deglutition disorders' | 104,044 |
| #25 **Dysphagia** 2 | (swallow* OR deglutition OR oropharyngeal) AND (problem* OR disorder* OR impairment* OR difficult* OR dysphagia) OR dysphagia | 130,796 |
| #26 **Outcome** | #24 OR #25 | 135,357 |
| #27 **Search** | #23 AND #26 | 1,266 |
