# Supplementary material for: Dental Factors Associated With Oropharyngeal Dysphagia in Institutionalised Older Adults: A Systematic Review
Source: Gerodontology. 2025 Dec 18;43(2):159–71. doi: 10.1111/ger.70034 (PMC13140015; doi:10.1111/ger.70034)
Supplement: Supplementary file 2 — Appendix S2: Reasons for exclusion of full‐text articles. [file GER-43-159-s002.docx]

APPENDIX 2

Reasons for exclusion of full-text articles

| Nº | Year | Article | Status | Reason for Exclusion |
| --- | --- | --- | --- | --- |
| 1 | 1995 | Xerostomia, xerogenic medications and food avoidances in selected geriatric groups | Excluded | No information on whether they are institutionalized or not; there is no group division. |
| 2 | 2002 | Oral health and nutritional status in a group of geriatric rehabilitation patients. | Excluded | Assessment of hospitalized patients with a health condition, living in the community, or receiving home care. |
| 3 | 2004 | Effect of oral function on nutritional improvement in nursing home residents | Excluded | Assessment of hospitalized patients with a health condition, living in the community, or receiving home care. |
| 4 | 2004 | [Care assessment related to dysphagia in the frail elderly]. | Included |  |
| 5 | 2005 | Nutritional status in cognitively intact older people receiving home care services - A pilot study | Excluded | Assessment of hospitalized patients with a health condition, living in the community, or receiving home care. |
| 6 | 2006 | Denture use, malnutrition, frailty, and mortality among older women living in the community | Excluded | Assessment of hospitalized patients with a health condition, living in the community, or receiving home care. |
| 7 | 2008 | A study of factors influenced by swallowing among chronic stroke patients | Excluded | Assessment of hospitalized patients with a health condition, living in the community, or receiving home care. |
| 8 | 2008 | Intervention study of exercise program for oral function in healthy elderly people. | Excluded | Assessment of hospitalized patients with a health condition, living in the community, or receiving home care. |
| 9 | 2008 | [6/6-Oral hygiene and deglutition disorders]. | Excluded | No access to the full article or corresponds only to an abstract for presentation or case report. |
| 10 | 2010 | Factors influencing oral health-related quality of life (OHRQoL) among the frail elderly residing in the community with their family. | Excluded | Assessment of hospitalized patients with a health condition, living in the community, or receiving home care. |
| 11 | 2012 | Associations between chewing and swallowing problems and physical and psychosocial health status of long-term care residents in taiwan: a pilot study. | Included |  |
| 12 | 2013 | Factors associated to suggestive signs of oropharyngeal dysphagia in institutionalized elderly women. | Included |  |
| 13 | 2013 | Relation between oral health, swallowing symptoms self-reported and frailty in Brazilian elderly | Excluded | No information on whether they are institutionalized or not; there is no group division. |
| 14 | 2013 | Interrelationship of oral health status, swallowing function, nutritional status, and cognitive ability with activities of daily living in Japanese elderly people receiving home care services due to physical disabilities. | Excluded | Assessment of hospitalized patients with a health condition, living in the community, or receiving home care. |
| 15 | 2013 | Salivary tests associated with elderly people's oral health. | Included |  |
| 16 | 2013 | The relationship between prosthetic status and the Geriatric Oral Health Assessment Index in a group of institutionalized elderly of an Indian city: a cross-sectional study. | Excluded | The information is grouped, with no exclusive assessment of swallowing. |
| 17 | 2015 | High prevalence of colonization of oral cavity by respiratory pathogens in frail older patients with oropharyngeal dysphagia. | Excluded | Assessment of hospitalized patients with a health condition, living in the community, or receiving home care. |
| 18 | 2015 | Functional relationship between risk factors of assessment of power with dysphagia in institutionalized elderly | Excluded | There is no group division regarding their oral condition." |
| 19 | 2015 | Prognosis-related factors concerning oral and general conditions for homebound older adults in Japan. | Excluded | There is no group division regarding their oral condition." |
| 20 | 2016 | Swallowing Function and Nutritional Status in Japanese Elderly People Receiving Home-care Services: A 1-year Longitudinal Study. | Excluded | Assessment of hospitalized patients with a health condition, living in the community, or receiving home care. |
| 21 | 2016 | Oral Health Status of Older Adults in Sweden Receiving Elder Care: Findings From Nursing Assessments. | Excluded | Assessment of hospitalized patients with a health condition, living in the community, or receiving home care. |
| 22 | 2017 | Relation between locomotion and oral/swallowing function in frail elderly patients | Excluded | No access to the full article or corresponds only to an abstract for presentation or case report. |
| 23 | 2017 | Factors related to skeletal muscle mass in the frail elderly. | Excluded | Assessment of hospitalized patients with a health condition, living in the community, or receiving home care. |
| 24 | 2017 | Posterior teeth occlusion and dysphagia risk in older nursing home residents: a cross-sectional observational study. | Included |  |
| 25 | 2017 | The Prevalence of Oropharyngeal Dysphagia in Danish Patients Hospitalised with Community-Acquired Pneumonia. | Excluded | Assessment of hospitalized patients with a health condition, living in the community, or receiving home care. |
| 26 | 2017 | Indicators of Dysphagia in Aged Care Facilities. | Excluded | There is no group division regarding their oral condition. |
| 27 | 2018 | Occlusal Support, Dysphagia, Malnutrition, and Activities of Daily Living in Aged Individuals Needing Long-Term Care: A Path Analysis | Excluded | Assessment of hospitalized patients with a health condition, living in the community, or receiving home care. |
| 28 | 2018 | Tooth loss, swallowing dysfunction and mortality in Japanese older adults receiving home care services | Excluded | Assessment of hospitalized patients with a health condition, living in the community, or receiving home care. |
| 29 | 2018 | Association between oropharyngeal dysphagia, oral functionality, and oral sensorimotor alteration. | Included |  |
| 30 | 2018 | Influence of dental factors on oropharyngeal dysphagia among recipients of long-term care. | Included |  |
| 31 | 2018 | Decreased cognitive function is associated with dysphagia risk in nursing home older residents. | Included |  |
| 32 | 2020 | Oral Management in Rehabilitation Medicine: Oral Frailty, Oral Sarcopenia, and Hospital-Associated Oral Problems. | Excluded | **Assessment of hospitalized patients with a health condition, living in the community, or receiving home care.** |
| 33 | 2020 | Association between oral and swallowing function and food items in elderly people at nursing homes | Excluded | No access to the full article or corresponds only to an abstract for presentation or case report. |
| 34 | 2020 | Oral Factors Associated with Swallowing Function in Independent Elders. | Excluded | Assessment of hospitalized patients with a health condition, living in the community, or receiving home care. |
| 35 | 2020 | Prevalence and risk factors of dysphagia among nursing home residents in eastern China: a cross-sectional study. | Included |  |
| 36 | 2020 | Masticatory function in nursing home residents: Correlation with the nutritional status and oral health-related quality of life. | Excluded | The information is grouped, with no exclusive assessment of swallowing. |
| 37 | 2021 | Impact of Oral and Swallowing Function on the Feeding Status of Older Adults in Nursing Homes. | Excluded | The information is grouped, with no exclusive assessment of swallowing. |
| 38 | 2021 | Number of teeth, denture wearing and cognitive function in relation to nutritional status in residents of nursing homes | Excluded | There is no direct comparison between oral health and dysphagia. |
| 39 | 2021 | Associations of poor oral health with frailty and physical functioning in the oldest old: results from two studies in England and Japan. | Excluded | Assessment of hospitalized patients with a health condition, living in the community, or receiving home care. |
| 40 | 2021 | Tongue cleaning maintains respiratory function in older individuals: A 1-year randomised controlled trial. | Excluded | There is no direct comparison between oral health and dysphagia. |
| 41 | 2021 | Prosthetic rehabilitation status, dental prosthesis functionality and masticatory function in nursing home residents. | Included |  |
| 42 | 2021 | Prevalence and Biological Correlates of Oropharyngeal Dysphagia in Outpatients of a Geriatric Evaluation Clinic: A Brief Report. | Excluded | No access to the full article or corresponds only to an abstract for presentation or case report. |
| 43 | 2023 | Relationship of occlusal status with health-related quality of life among older adults in long-term care facilities | Included |  |
| 44 | 2023 | Prosthetic rehabilitation status, dental prosthesis functionality and masticatory function in nursing home residents. | Excluded | The information is grouped, with no exclusive assessment of swallowing. |
